# Sagittal Alignment Reciprocal Changes After Thoracolumbar/Lumbar Anterior Vertebral Body Tethering

**DOI:** 10.3390/jcm15020447

**Published:** 2026-01-06

**Authors:** Taha Furkan Yağcı, Serkan Bayram, Murat Korkmaz, Şahin Karalar, Adem Bayraktar, Gökhan Bayrak, Turgut Akgül

**Affiliations:** 1 Department of Orthopedics and Traumatology, Muş State Hospital, 49250 Muş, Türkiye; 2 Department of Orthopedics and Traumatology, İstanbul Faculty of Medicine, İstanbul University, 34093 İstanbul, Türkiye; 3 Department of Orthopedics and Traumatology, Faculty of Medicine, Medipol University, 34815 İstanbul, Türkiye; 4 Department of General Surgery, İstanbul Faculty of Medicine, İstanbul University, 34093 İstanbul, Türkiye; 5 Department of Physiotherapy and Rehabilitation, Faculty of Health Sciences, Muş Alparslan University, 49250 Muş, Türkiye; fztgokhanbayrak@gmail.com

**Keywords:** lumbar anterior vertebral body tethering, sagittal alignment, adolescent idiopathic scoliosis

## Abstract

**Background/Objective:** The anterior vertebral body tethering (AVBT) technique, which preserves spinal mobility and avoids possible fusion problems in adolescent idiopathic scoliosis (AIS) patients, continues to be increasingly used in spine surgery. The study aims to report the early-to-early-mid postoperative radiological results of thoracolumbar/lumbar AVBT on sagittal alignment, and the second aim is to compare AVBT with selective thoracic fusion (STF) and non-selective fusion (NSF) groups in AIS patients. **Methods:** Patients with a diagnosis of AIS were retrospectively evaluated in the study. All patients were categorized into three groups based on the surgical technique performed: AVBT (n = 17), NSF (n = 19), and STF (n = 15). The major curvature degree, coracoid height difference (CHD), sacral slope (SS), pelvic tilt (PT), pelvic incidence (PI), lumbar lordosis (LL), thoracic kyphosis (TK), cervical lordosis (CL), C7 tilt, sagittal vertical axis (SVA), T1 pelvic angle (TPA), and T1 spinopelvic inclination (T1SPI) were measured for radiological comparison. Scoliosis Research Society-22 (SRS-22) and Oswestry Disability Index (ODI) scores were used at the final follow-up for functional evaluation. **Results:** The T1SPI value of the NSF group was significantly higher than the STF group in the final follow-up (*p* = 0.033). The mean decrease of 8.85 ± 7.85 units in the final follow-up value compared to the postoperative CHD value of the patients in the AVBT group was found to be significant (*p* = 0.028). Statistically significant differences were found between preoperative and the first postoperative CL and TPA measurements (*p* = 0.001 and *p* = 0.042, respectively), as well as between preoperative and final follow-up CL measurements in the AVBT group (*p* = 0.001). No statistically significant differences were observed between the groups in CHD, SS, PT, PI, LL, TK, CL, C7 tilt, SVA, and TPA values (*p* > 0.05); similarly, the SRS-22 and ODI scores did not differ significantly among the groups (*p* > 0.05). **Conclusions:** Thoracolumbar/lumbar AVBT surgery led to significant improvements in shoulder asymmetry and cervical lordosis of AIS patients in the early to early-mid postoperative period. However, compared with spinal fusion techniques, thoracolumbar/lumbar AVBT did not demonstrate superiority in functional scores or sagittal parameters. The mid- to long-term benefits of thoracolumbar/lumbar AVBT remain uncertain and require further investigation.

## 1. Introduction

Posterior spinal fusion (PSF) has proven effective in controlling the progression of deformities in scoliosis treatment. However, it has limitations such as reduced spinal movement, growth restriction, and the potential for adjacent segment disease [1,2]. Anterior vertebral body tethering (AVBT) surgery is a promising surgery for patients with great growing potential as a growth-sparing method, and an earlier report underscores the satisfactory outcomes associated with this technique [3]. Also, the first indication for AVBT was pronounced for thoracic deformity, which was recently published and showed that this technique is useful in lumbar or double major curves [4]. According to comparative studies, PSF showed great success in correcting coronal deformity against AVBT with a lower complication rate [5].

Clinical studies reported the results of AVBT, which were focused on mobility and coronal deformity correction. The studies showed that patients with AVBT have higher clinical mobility, which was also proved radiologically [6,7]. In the thoracolumbar and lumbar regions, motion is more important than in the thoracic region. The lumbar AVBT results published by Trobisch et al. [8] are satisfactory and provide clinical results, including radiological measurements, with acceptable complication rates.

Sagittal spinal alignment is fundamental to spinal biomechanics and long-term clinical outcomes, functioning as a key determinant of posture, energy expenditure, and compensatory spinal–pelvic movements. Global sagittal parameters, including sagittal vertical axis (SVA), pelvic tilt (PT), and T1-pelvic angle (TPA), have been shown to correlate strongly with health-related quality of life, pain, and functional disability in spine deformity populations [9]. Disturbances in the cervical, thoracic, and lumbar curvatures can induce compensatory mechanisms above or below the affected region, which may contribute to junctional problems, muscle fatigue, gait inefficiency, and long-term imbalance [10]. In the context of adolescent idiopathic scoliosis (AIS), the significance of sagittal alignment is particularly improved, especially in relation to growth-modulating interventions such as AVBT. Insufficient sagittal correction can potentially constrain the advantages associated with spinal mobility and make patients more susceptible to subsequent mechanical complications [11]. Given that AIS is a three-dimensional deformity and that recent reviews indicate neglecting sagittal alignment in surgical planning, regardless of coronal correction, may lead to long-term imbalance or functional impairment, measuring global and regional sagittal parameters becomes especially critical in patients undergoing AVBT [12].

Considering the limited number of studies specifically evaluating sagittal and global alignment following thoracolumbar/lumbar AVBT in the literature, a detailed radiographic assessment of these parameters is essential to understand the biomechanical impact of different surgical strategies in AIS [8,13]. Therefore, the primary aim of our study is to report the early-to-early-mid radiological results of thoracolumbar/lumbar AVBT on sagittal parameters. Our second aim is to compare AVBT functionally and radiologically with selective thoracic fusion (STF), which preserves lumbar motion, and non-selective fusion groups (NSF), which restrict lumbar motion.

## 2. Methods

### 2.1. Study Design

The study was designed as a retrospective analysis conducted at a single center. This study was conducted within the Department of Orthopedics and Traumatology of İstanbul University, İstanbul Faculty of Medicine, and the interventions, examinations, and data collection performed on the patients were carried out in this institution. Ethics committee approval was received from the İstanbul University Faculty of Medicine Clinical Research Ethics Committee (protocol code 1984626, approval date 8 August 2023).

### 2.2. Patients

This study included patients who underwent surgery for AIS using thoracolumbar/lumbar AVBT, STF, and NSF techniques between 2018 and 2023, with the surgical technique chosen based on the patients’ Lenke, Risser, and Sanders categories by a specialized, well-experienced surgeon. The inclusion criteria were having been operated on with the diagnosis of AIS and being 10–25 years old. Exclusion criteria included the use of the thoracic AVBT technique (lowest instrumented vertebra [LIV] L1 and above), patients presenting with congenital anomalies, including fusion and segmentation deformities of the vertebrae, patients diagnosed with syndromic conditions or neuromuscular disorders, and neurological examination findings indicating sensory or motor deficits. Furthermore, patients exhibiting a spinal curvature exceeding 60 degrees, those who had undergone prior spinal surgeries, as well as individuals who had previously experienced surgical interventions within the thoracic cavity, a previous history of vertebral infections, and any intraspinal pathologies necessitating neurosurgical monitoring or intervention were also excluded.

Fifty-one patients were included in the study. Patients were separated into three groups. The groups were

**Figure 1:**
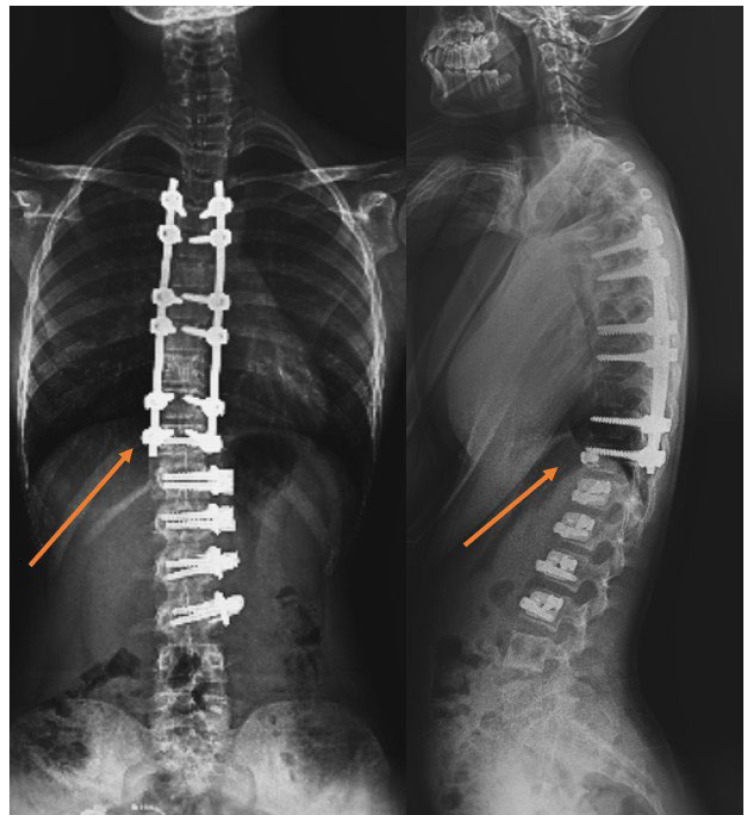
Example of a case with hybrid fusion; the vertebra marked with an arrow is called the transitional vertebra.

▪ In patients who underwent tethering, those with an LIV level at L2, L3, and L4 formed the thoracolumbar/lumbar AVBT group (Group 1). Patients who underwent AVBT in the thoracolumbar region and hybrid surgery, combining AVBT with thoracic fusion, were included in the AVBT group (Figure 1). The majority of thoracolumbar/lumbar AVBT cases underwent hybrid fusion procedures (9 out of 17, 52.9%).
▪ In patients undergoing fusion, those with an LIV level at L2, L3, and L4 formed the NSF group (Group 2).
▪ In patients undergoing fusion, those with an LIV level of L1 and superior (T10, T11, and T12) formed the STF group (Group 3).

The power ratio of the sample was calculated based on a previous study’s lumbar curve degree, and the effect size of this study was determined as d = 0.77 (large) [5]. According to the power analysis of the F-tests to determine a difference in thoracal curvature of the groups, assuming that we could achieve a similar effect size (d = 0.77), a level of 0.05, and a power of 80%, the a priori calculated sample size was at least 36 patients (at least 12 patients for each group). When the post hoc power analysis based on the lumbar lordosis results from the study was examined, a large effect size (d = 0.61) was found between the groups for lumbar curve angle. For this effect size, our study had 97% power at a 95% confidence level at final assessments.

### 2.3. Outcome Measures

Standing spinal anteroposterior and lateral radiographs of all patients were taken. During X-ray imaging, all full-spine standing radiographs were obtained using a standardized protocol to ensure sagittal and coronal alignment measurements. Patients were positioned in a standardized X-ray imaging position: an upright stance with knees fully extended, feet fully weight-bearing, and feet shoulder-width apart. Arm positioning followed the current recommendations for sagittal balance assessment: patients placed both hands crossed at the shoulders to prevent overlapping of the thoracic spine. The head was maintained in a forward-looking, neutral gaze to avoid unintended cervical flexion or extension. Radiographs were acquired in a single long-cassette anteroposterior and lateral format, ensuring visualization from the skull base to the femoral heads. All images were obtained using the same radiographic equipment and acquisition settings within our institution to minimize variability. All radiographic measurements were performed using our institution’s Picture Archiving and Communication System (PACS), which provides calibrated digital tools for accurate angular and linear measurements.

The anteroposterior radiograph evaluated the type of scoliosis, curvature pattern, balance of the spine and trunk, and shoulder asymmetry. After the anteroposterior and lateral radiographs, lateral bending and traction radiographs were taken. The major degree of curvature and the vertebrae causing the major curvature were determined for each patient. Neutral and end vertebrae were defined for each curvature. Side bending and traction radiographs were also evaluated, and the Lenke classification was made, considering non-structural curvatures. A preoperative plan was made by determining the levels to be instrumented during surgery. Anteroposterior radiographs of the wrist were taken to evaluate skeletal maturity.

For all patients, Sanders’s skeletal maturity staging, Risser signs, and Lenke classification were determined from preoperative radiographs regarding age, gender, and maturity assessment.

**Figure 2:**
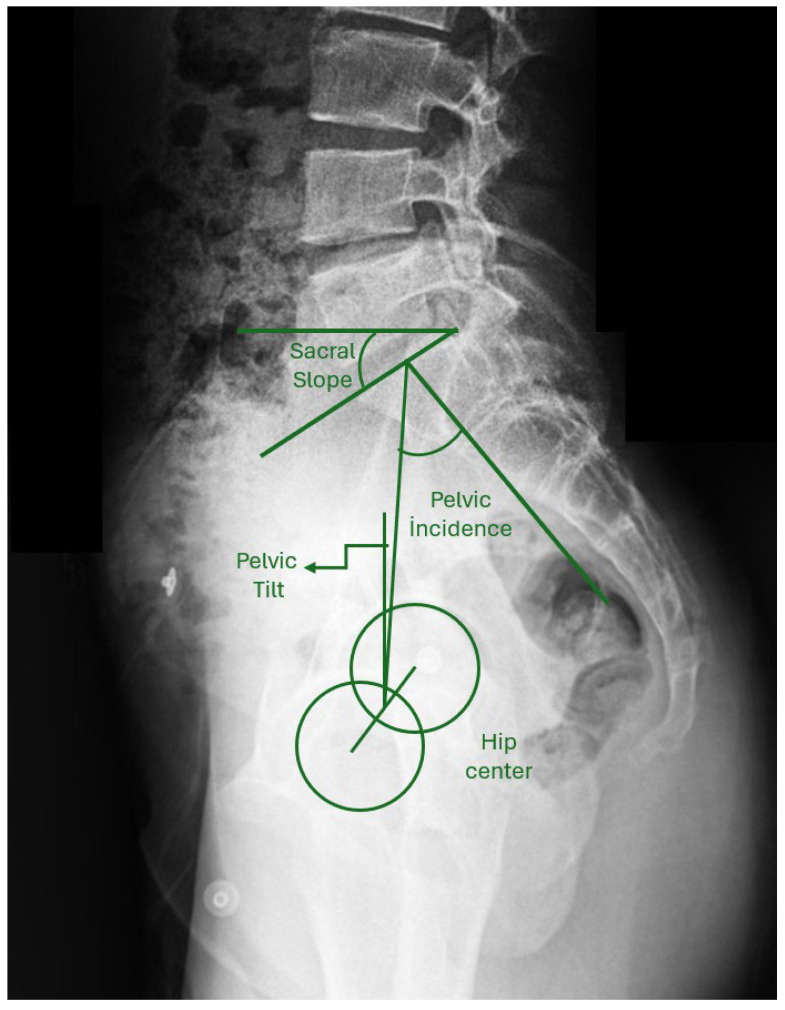
Sacral slope, pelvic tilt, and pelvic incidence (pelvic parameters).

**Figure 3:**
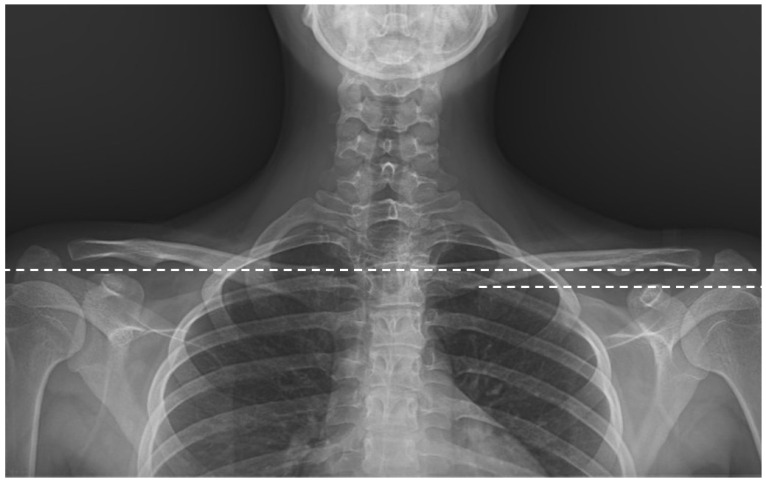
CHD is the height difference in millimeters between the horizontal lines passing through the upper margin of each coracoid process.

**Figure 4:**
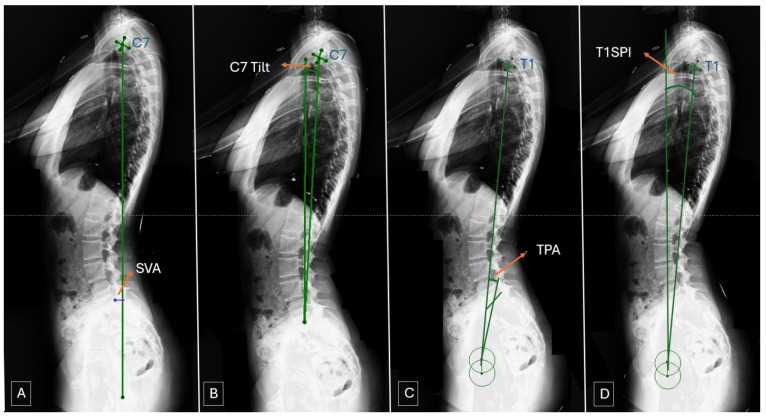
Global sagittal parameters’ measurement ((**A**): SVA; (**B**): C7 Tilt; (**C**): TPA; (**D**): T1SPI).

The degree of major curvature was measured. Cervical lordosis (CL), thoracic kyphosis (TK, T4-12), and lumbar lordosis (LL) were measured. Pelvic parameters were evaluated by measuring sacral slope (SS), pelvic incidence (PI), and PT (Figure 2). Coracoid height distance (CHD) was measured to evaluate shoulder asymmetry (Figure 3). To evaluate global sagittal parameters, the SVA, the tilt of the C7 vertebra (C7 tilt), the TPA, and the T1 spinopelvic inclination (T1SPI) were measured (Figure 4). All measurements were repeated three times for each of the three patient groups: preoperatively, on the first postoperative day, and at final follow-up radiographs.

Tether breakage was defined as loss of correction of more than 5 degrees between screws in consecutive measurements, as described in the literature [14].

Radiographic quantitative measurements were independently assessed by two spinal surgeons, and the results were averaged. For non-quantitative data such as Sanders’s maturity staging system, the Risser sign, and the Lenke classification, consensus was sought between two observers; in cases where no consensus was reached, a decision was made by consulting a third observer.

To evaluate postoperative functional scores, a questionnaire was administered to the patients for Scoliosis Research Society-22 (SRS-22) and Oswestry Disability Index (ODI) scores. The SRS-22 questionnaire consists of five subgroups: pain, self-image/appearance, degree of function/activity, mental health, and treatment satisfaction. The total score ranges from 1 to 5 points, with a higher score indicating a better patient quality of life [15]. The ODI score is a questionnaire that measures how much low back pain affects daily activities and is evaluated through 10 questions [16,17]. For each question, the situation that causes the least pain is evaluated by scoring between 0 and 5 points, with 0 points for the most severe pain and 5 points for the least severe pain. All points applied by the patient to the survey are added up, and the percentage is calculated.

### 2.4. Statistical Analyses

IBM SPSS software (IBM SPSS Statistics for Windows, Version 26.0. Armonk, NY, USA) was used for statistical analysis. Descriptive statistical methods (mean, standard deviation, median, frequency, percentage, minimum, and maximum) were used when evaluating the study data. The suitability of quantitative data for normal distribution was tested using the Shapiro–Wilk test and graphical analysis. While one-way analysis of variance (ANOVA) was used to compare normally distributed quantitative variables between more than two groups, Bonferroni-corrected binary evaluations were used when the variances were homogeneous. The Games-Howell test was used when the variances were not homogeneous to determine which group the significance originated from. In comparisons of non-normally distributed quantitative variables between more than two groups, the Kruskal–Wallis test was used, and the Dunn-Bonferroni test was used to determine which group caused the significance. The Friedman test was used for intragroup comparisons of quantitative variables that did not show normal distribution, and the Wilcoxon signed-rank test with Bonferroni correction was used to evaluate pairwise comparisons. The Wilcoxon signed-rank test was used for intragroup comparisons of quantitative variables that did not show normal distribution. The Fisher-Freeman-Halton test was used to compare qualitative data. Statistical significance was accepted as *p* < 0.05.

## 3. Results

### 3.1. Group Demographics

The study included 51 patients’ data: 17 in the AVBT group, 19 in the NSF group, and 15 in the STF group. Of the patients, 19.6% (n = 10) were male, and 80.4% (n = 41) were female. The ages of the study participants ranged between 10.52 and 25.07 years, and the average age was 15.57 ± 2.97 years. Demographic data of the groups, the Risser sign, and the Lenke classification are summarized in Table 1.

The average AVBT instrument level in the study was 5.23 ± 1.3. Table 2 summarizes the age, gender, instrument levels, Sanders’s skeletal maturity stage, Risser sign, and Lenke classification of the patients in the AVBT group.

The average follow-up period was 22.7 months in the AVBT group, 23.27 months in the STF group, and 23.33 months in the NSF group.

### 3.2. Preoperative Radiographic Parameters

No statistically significant difference was detected between the preoperative major curvature degrees, the CHD, SS, PT, PI, LL, TK, SVA, CL, C7 Tilt, TPA, and T1SPI measurements of the patients according to the groups in preoperative radiographic (*p* > 0.05). The comparison of measurements between groups is summarized in Table 3.

### 3.3. Early Postoperative Results

In the first postoperative radiograph, CHD, SS, PT, PI, TK, LL, SVA, CL, C7 Tilt, TPA, and T1SPI measurements did not show a statistically significant difference (*p* > 0.05). Table 4 summarizes the intergroup comparison of the first postoperative radiographic measurements.

### 3.4. Final Follow-Up Results

No statistically significant difference was detected in CHD, SS, PT, PI, LL, TK, SVA, CL, C7 Tilt, and TPA measurements in the final follow-up radiography measurements (*p* > 0.05). A statistically significant difference was found between the T1SPI values in the final follow-up radiography measurements of the patients according to the groups (*p* = 0.033). Intergroup comparison of final follow-up radiographic measurements is summarized in Table 5.

### 3.5. Intragroup Longitudinal Changes (AVBT Group)

A statistically significant difference was detected between first and final follow-up CHD measurements (*p* = 0.028). A statistically significant difference was detected between preoperative and final postoperative CL measurements (*p* = 0.001). A statistically significant difference was detected between preoperative and first postoperative TPA measurements (*p* = 0.042). In the analysis comparing the preoperative, postoperative, and final follow-up radiographs of patients in the AVBT group, no statistically significant difference was found in SS, PT, PI, LL, TK, SVA, C7 tilt, or T1SPI measurements (*p* > 0.05). The analysis performed within the AVBT group is summarized in Table 6.

### 3.6. Functional Outcomes

There was no statistically significant difference between the groups in SRS-22 total score and sub-scores of pain, image, function, mental health, and satisfaction with treatment (*p* > 0.05). No statistically significant difference was found between the patients’ ODI scores according to the groups (*p* > 0.05). Table 7 summarizes the comparison of functional scores between groups.

### 3.7. Complications

**Figure 5:**
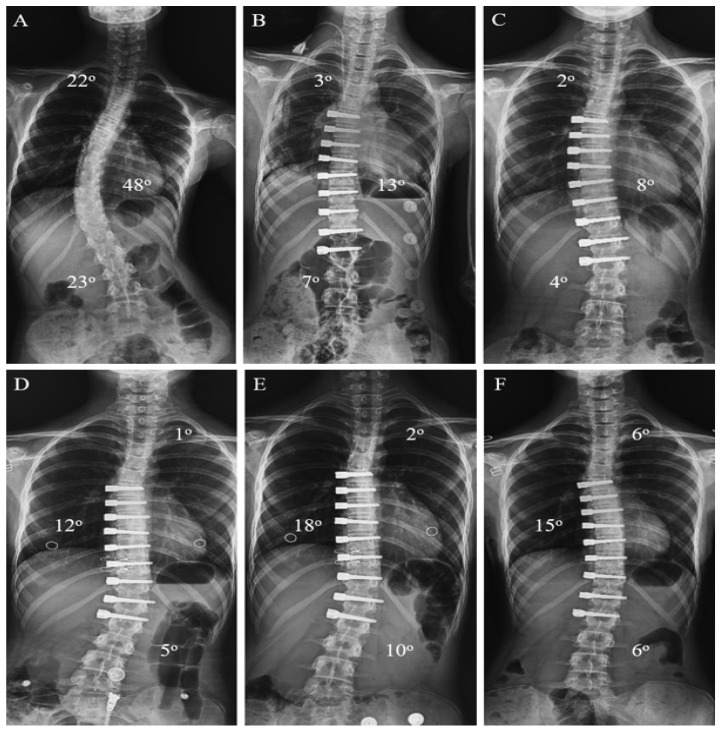
Anteroposterior radiograph of a patient who developed overcorrection complications. ((**A**): Preoperative radiography of a patient aged 11 years and 9 months; (**B**): First postoperative day radiography after AVBT surgery; (**C**): Postoperative sixth-month radiography; (**D**): One-year postoperative radiograph; (**E**): 1.5-year postoperative radiograph, patients aged 13 years and 3 months; (**F**): Eighth month of postoperative radiography after revision surgery, patient aged 14 years).

No perioperative complications related to intraoperative anesthesia and surgery were observed. We identified suspicion of tether breakage in just one patient. But we monitored the patient, and we did not perform revision surgery because the patient reached maturity during the follow-up period and did not progress in curvature. Additionally, overcorrection occurred in one patient in the AVBT group. T6-L2 levels were instrumented with AVBT in 11 years and 9 months old female patient, Sanders 6, Risser stage 1, right major curvature 48 degrees in the preoperative evaluation, Lenke 1 patient. After the surgery, the major curvature dropped to 13 degrees. However, 1.5 years after surgery, the measurements were recorded as 18 degrees of left major curvature, and the revision surgery was planned and performed. Her previous surgery was performed with mini-open thoracotomy and lumbotomy. Using the prior incision, the tether was cut between T11-12 and T12-L1. After the revision, the eighth-month radiograph showed that the curvature had decreased to 15 degrees (Figure 5).

## 4. Discussion

The main finding of this study is that although thoracolumbar/lumbar AVBT does not allow direct correction in the sagittal plane, it yields results on sagittal parameters similar to those of the fusion groups. In addition, although thoracolumbar/lumbar AVBT preserves motion in the surgical technique, functional results were found to be similar to those of fusion groups.

When sagittal parameters were evaluated, Trobisch et al. [8] published the results of lumbar AVBT in their study with 35 patients and found that TK, LL, SVA, PT, and PI parameters did not change after AVBT. In another study, which included 86 patients who underwent AVBT and examined sagittal parameters during a 2-year follow-up period, they found that the average TK increased from 28.3 degrees to 33 degrees, the average LL did not change, the average PT decreased from 9.4 degrees to 7.4 degrees, and that AVBT did not have a kyphotic effect on the LL [13]. Baroncini et al. [18], in a review in which nine studies were evaluated, reported no significant change in TK and LL values. The meta-analysis conducted by Mariscal et al. [19], including 12 studies, found that TK and LL did not change after AVBT. Our study found that SS, PT, PI, LL, and TK values did not show a statistically significant change in the pre- and postoperative comparison in the AVBT group. The consistency of our results with prior literature supports the idea that AVBT exerts a largely neutral influence on sagittal alignment. This suggests that tethering’s gradual correction mechanism allows coronal correction without imposing abnormal forces on sagittal morphology. By maintaining the lumbar lordosis–thoracic kyphosis relationship without inducing compensatory shifts in pelvic or thoracic parameters, AVBT appears biomechanically compatible with the preservation of physiological sagittal alignment.

Previous studies have not addressed changes in CL in patients undergoing AVBT surgery, leaving the influence of tethering on proximal sagittal alignment insufficiently characterized in the current literature. Our study detected a significant increase in cervical lordosis value after surgery, which may reflect a compensatory proximal adjustment aimed at maintaining global sagittal harmony in response to anterior thoracic modulation. To our knowledge, no study has evaluated global sagittal parameters in patients who underwent AVBT surgery, except Baroncini’s study, which shows that SVA decreased from 4.5 mm to 3.6 mm on average after AVBT [13]. Our study found that SVA, C7 tilt, and T1SPI values did not show statistically significant changes in the pre- and postoperative evaluation of patients who underwent AVBT surgery, suggesting that AVBT largely preserves global sagittal alignment and does not induce maladaptive compensatory mechanisms despite localized adjustments in cervical and thoracic regions.

In patients who underwent AVBT, the early postoperative increase observed in TPA suggested a transient alteration in global sagittal alignment. However, this change did not persist over time, as final follow-up assessments demonstrated that TPA values effectively returned to preoperative levels. This pattern indicates that the initial postoperative variation likely reflects short-term adaptive or mechanical factors rather than a sustained sagittal imbalance attributable to the surgical procedure. The transient elevation in TPA supports the interpretation that early postoperative mechanical or muscular adjustments occur following tethering but are self-limiting. The normalization of TPA highlights the capacity of patients’ postural control systems to re-establish equilibrium, confirming that AVBT does not impose a sustained sagittal imbalance despite these initial adaptive fluctuations.

Pehlivanoğlu et al. [4] reported that AVBT in thoracolumbar curves provided significantly better lumbar range of motion and forward and lateral flexibility, resulting in a better SRS-22 score than patients who underwent thoracolumbar PSF. Their study highlights the theoretical advantage of motion preservation, which is one of the core rationales of growth-modulating techniques. In a meta-analysis comparing the mid-term results of 10 AVBT and 14 PSF studies with Lenke 1 and 2 patients when evaluated as a functional score, SRS-22 scores were reported to be similar for the two groups [20]. In a study conducted by matching 237 AVBT patients with 237 PSF patients and comparing their preoperative and postoperative SRS-22 scores during a 2-year follow-up period, they reported that the mental health sub-score was lower in the AVBT group before surgery [5]. No difference was detected between ODI scores and the Total SRS-22 score and the sub-scores of Pain, Image, Function, Mental Health, Satisfaction with Treatment, STF, NSF, and AVBT groups. The results indicate that AVBT does not provide a measurable advantage in early functional outcomes compared with fusion, despite its intended motion-preserving properties. However, the SRS-22 score, utilized to evaluate function, does not specifically assess spinal mobility; therefore, postoperative scores may not fully capture the potential benefits of the AVBT technique in this study. Historically, scoliosis treatment predominantly relied on spinal fusion, with little consideration of spinal mobility in existing functional assessment scales. However, as the AVBT technique and preservation of spinal mobility have gained prominence in recent years, there is an emerging need to develop a novel functional scale to specifically evaluate spinal mobility.

There are many radiological measurement parameters regarding shoulder asymmetry. An earlier study has reported that the radiological measurements with the highest correlation with clinically detected shoulder asymmetry are CHD, clavicle angle, and clavicle–first rib intersection difference [21]. Shoulder asymmetry in patients undergoing thoracolumbar AVBT surgery has yet to be discussed in the literature. We did not detect any difference between the preoperative and postoperative mean CHD values in the AVBT group. Still, we found that the mean CHD decreased at the final follow-up compared to the first postoperative radiograph. The reason for this may be that the trunk finds its coronal balance and achieves shoulder balance due to the correction of the curvature over time. As the tether modulates the thoracolumbar curve and stabilizes trunk alignment, improved load distribution and thoracic leveling likely allow the shoulder girdle to reposition naturally, emphasizing the relationship of coronal balance and shoulder symmetry.

In patients undergoing AVBT surgery, tether breakage has been documented with highly variable rates, ranging from 0% (0/10) to 73% (22/30) in previous studies [4,22]. A systematic review conducted by Zhang et al., which encompassed 25 studies, revealed a tether breakage rate of 21.3% [23]. Meanwhile, Mariscal et al. published a meta-analysis of 12 studies, finding a lower tether breakage rate of 5.9% [19]. In our study, we identified suspicion of tether breakage in just one patient. This finding is less common in the literature, likely due to our use of double-row tethers (13/17) in the majority of cases and the fact that the patients were primarily undergoing hybrid fusion procedures (9/17). Additionally, most patients in our study were at Sanders stage six or higher at the time of surgery (11/17), which suggests that the tether breakage rate may have remained low due to the patients being close to full skeletal maturity. It is important to note that our results stem from short-term follow-up, and tether breakage rates may change with longer-term assessments.

The primary limitations of this study include the absence of randomization and its retrospective design. As lumbar AVBT is a novel surgical technique, only a limited number of cases have been performed in the thoracolumbar region, leading to the inclusion of only a few pure AVBT cases. Consequently, more than half of the AVBT group underwent hybrid procedures combining thoracolumbar/lumbar AVBT with thoracic fusion (9 of 17 patients, 52.9%), thereby reducing group homogeneity and possibly influencing functional and mobility-related outcomes. Additionally, thoracolumbar flexion-extension and lateral flexion mobility were not evaluated, which may have obscured potential differences between groups. Although the study’s follow-up duration allowed an evaluation of early to early-mid postoperative changes, it was insufficient to determine mid-term outcomes regarding the lasting sagittal balance and functional superiority. Finally, the single-center setting restricts the generalizability of the findings.

## 5. Conclusions

Our study is the first to report findings on shoulder asymmetry following thoracolumbar/lumbar AVBT surgery, and it indicates that this technique did not demonstrate superiority over fusion groups. Patients who underwent the AVBT technique showed significant improvement in shoulder asymmetry over time, attributable to effective correction of spinal curvature. Additionally, our assessment of early-to-early-mid postoperative functional scores reveals no significant advantage for the AVBT group compared to the fusion groups. Cervical lordosis demonstrated a remarkable improvement following AVBT surgery, particularly compared with preoperative measurements. Although the average follow-up period of 22.7 months in the thoracolumbar/lumbar AVBT cohort provides important knowledge of early-to-early-mid postoperative outcomes, further research with extended follow-up is necessary to reveal the durability of these surgical outcomes and their effects on mid- to long-term patient function and mobility. Overall, the findings indicate a significant improvement in cervical lordosis following AVBT surgery, while sagittal parameters and functional outcomes demonstrate similar outcomes between fusion and thoracolumbar/lumbar AVBT procedures.

## Figures and Tables

**Table 1:** Demographic characteristics of the groups.

Variables		Group 1 (AVBT, n = 17)	Group 2 (NSF, n = 19)	Group 3 (STF, n = 15)
		Mean ± SD	Mean ± SD	Mean ± SD
Age (year)		13.98 ± 2.34	16.81 ± 3.42	15.81 ± 2.24
		n (%)	n (%)	n (%)
Risser	Stage 0	4 (23.5)	1 (5.3)	0 (0.0)
	Stage I	2 (11.8)	0 (0.0)	0 (0.0)
	Stage II	0 (0.0)	1 (5.3)	0 (0.0)
	Stage III	1 (5.9)	1 (5.3)	1 (6.7)
	Stage IV	7 (41.2)	6 (31.6)	8 (53.3)
	Stage V	3 (17.6)	10 (52.6)	6 (40.0)
Lenke	Lenke I	8 (47.1)	3 (15.8)	11 (73.3)
	Lenke II	0 (0.0)	2 (10.5)	4 (26.7)
	Lenke V	9 (52.9)	9 (47.4)	0 (0.0)
	Lenke VI	0 (0.0)	5 (26.3)	0 (0.0)

AVBT: Anterior vertebral body tethering; NSF: Non-selective fusion; STF: Selective thoracic fusion; SD: Standard deviation.

**Table 2:** Characteristics of cases in the AVBT group.

Case	Age (Year)	AVBT Levels	Tethering	Posterior Fusion	Sanders Stage	Risser Stage	Lenke Classification
1	13.8	T10-L3	Double	No	4	0	5
2	11.9	T10-L3	Double	No	3	0	5
3	13.0	T10-L3	Double	No	5	4	5
4	11.9	T6-L2	Single	No	6	1	1
5	20.5	T11-L3	Double	No	8	5	5
6	16.0	T11-L3	Double	No	7	4	5
7	13.3	T10-L3	Double	No	7	4	1
8	16.7	T12-L4	Double	T5-12	8	5	1
9	11.5	T11-L3	Double	T4-T11	2	0	1
10	13.9	T12-L4	Single	T5-T12	4	3	5
11	15.0	T12-L3	Single	T2-T12	7	4	1
12	14.6	T12-L3	Double	T4-T12	7	4	1
13	14.7	T11-L3	Double	T5-T11	8	5	5
14	14.4	T12-L3	Double	T3-12	7	4	5
15	10.5	T11-L3	Single	T5-T11	3	0	1
16	12.1	T11-L3	Double	T2-T11	6	1	1
17	13.9	T12-L4	Double	No	7	4	5

AVBT: Anterior vertebral body tethering.

**Table 3:** Preoperative radiographic measurements of the groups.

Variables	Group 1 (AVBT, n = 17)	Group 2 (NSF, n = 19)	Group 3 (STF, n = 15)	*p*
	Mean ± SD	Mean ± SD	Mean ± SD	
CHD (mm)	16.33 ± 10.79	15.53 ± 12.47	9.82 ± 8.82	0.184
SS (°)	39.67 ± 4.22	35.22 ± 11.85	42.35 ± 9.67	0.187
PT (°)	8.71 ± 9.24	9.18 ± 9.98	7.35 ± 9.93	0.766
PI (°)	48.5 ± 11.21	42.92 ± 16.82	47.07 ± 14.03	0.404
LL (°)	51.94 ± 7.32	51.71 ± 11.56	54.39 ± 19.02	0.565
TK (°)	31.25 ± 10.37	32.11 ± 12.58	32.07 ± 13.51	0.944
SVA (°)	−3.08 ± 20	−0.72 ± 23.94	−3.24 ± 25.43	0.964
CL (°)	−1.37 ± 11.54	6.46 ± 11.09	−2.43 ± 18.39	0.149
C7 Tilt (°)	−2.03 ± 2.43	−2.87 ± 3.25	−1.95 ± 2.92	0.578
TPA (°)	3.74 ± 9.71	3.67 ± 7.36	2.8 ± 9.11	0.793
T1SPI (°)	−3.25 ± 1.31	−3.71 ± 2.76	−3.06 ± 4.26	0.819

AVBT: Anterior vertebral body tethering; NSF: Non-selective fusion; STF: Selective thoracic fusion; SD: Standard deviation; CHD: Coracoid Height Distance; SS: Sacral Slope; PT: Pelvic Tilt; PI: Pelvic Incidence; LL: Lumbar Lordosis; TK: Thoracic Kyphosis (T4-12); SVA: Sagittal Vertical Axis; CL: Cervical Lordosis; TPA: T1-Pelvic Angle; T1SPI: T1 Spinopelvic Inclination. Kruskal–Wallis Test.

**Table 4:** First postoperative radiographic measurements of the groups.

Variables	Group 1 (AVBT, n = 17)	Group 2 (NSF, n = 19)	Group 3 (STF, n = 15)	*p*
	Mean ± SD	Mean ± SD	Mean ± SD	
CHD (mm)	18.32 ± 10.93	14.56 ± 8.82	11.49 ± 8.22	^a^ 0.139
SS (°)	38.44 ± 8.96	36.22 ± 8.43	39.07 ± 8.94	^b^ 0.789
PT (°)	10.17 ± 8.66	9.4 ± 9.83	11.34 ± 9.32	^b^ 0.848
PI (°)	47.09 ± 16	45.53 ± 14.76	50.06 ± 11.71	^b^ 0.595
LL (°)	51.41 ± 10.47	51.09 ± 12.86	52.51 ± 11.16	^a^ 0.936
TK (°)	28.33 ± 6.18	33.32 ± 9.64	26.61 ± 8.49	^a^ 0.059
SVA (mm)	9.78 ± 25.12	3.01 ± 35.57	11.22 ± 25.22	^b^ 0.899
CL (°)	10.9 ± 6.48	7.23 ± 7.34	2.18 ± 20.98	^b^ 0.532
C7 Tilt (°)	3.48 ± 16.54	−2.15 ± 4.25	−0.44 ± 3.62	^b^ 0.318
TPA (°)	7.01 ± 8.84	5.06 ± 10.16	7.67 ± 8.58	^b^ 0.742
T1SPI (°)	−2.65 ± 2.71	−4.3 ± 2.8	5.26 ± 25.24	^b^ 0.097

AVBT: Anterior vertebral body tethering; NSF: Non-selective fusion; STF: Selective thoracic fusion; SD: Standard deviation; CHD: Coracoid Height Distance; SS: Sacral Slope; PT: Pelvic Tilt; PI: Pelvic Incidence; LL: Lumbar Lordosis; TK: Thoracic Kyphosis (T4-12); SVA: Sagittal Vertical Axis; CL: Cervical Lordosis; TPA: T1-Pelvic Angle; T1SPI: T1 Spinopelvic Inclination. ^a^ One-way ANOVA. ^b^ Kruskal–Wallis Test.

**Table 5:** Final follow-up radiographic measurements of the groups.

Variables	Group 1 (AVBT, n = 17)	Group 2 (NSF, n = 19)	Group 3 (STF, n = 15)	*p*
	Mean ± SD	Mean ± SD	Mean ± SD	
CHD (mm)	10.29 ± 8.37	10.31 ± 8.39	7.17 ± 6.61	^b^ 0.390
SS (°)	36.27 ± 6.81	37.04 ± 7.41	41.21 ± 6.72	^b^ 0.119
PT (°)	7.76 ± 10.26	9.79 ± 7.67	7.03 ± 9.74	^a^ 0.659
PI (°)	42.27 ± 12.05	46.2 ± 13.3	45.46 ± 16.66	^a^ 0.705
LL (°)	53.79 ± 13.58	54.91 ± 8.11	60.04 ± 8.67	^a^ 0.208
TK (°)	34.08 ± 8.98	33.76 ± 10.81	30.41 ± 8.95	^a^ 0.509
SVA (mm)	2.79 ± 32.96	−12.15 ± 26.45	−5.33 ± 23.94	^a^ 0.301
CL (°)	11.82 ± 9.4	15.48 ± 13.23	10.35 ± 12.48	^b^ 0.661
C7 Tilt (°)	−3.58 ± 6.99	−4.16 ± 4.87	−1.91 ± 3.14	^b^ 0.310
TPA (°)	4.6 ± 8.8	3.56 ± 8.04	4.3 ± 8.87	^a^ 0.943
T1SPI (°)	−3.98 ± 3.51	−5.67 ± 1.84	−3.11 ± 3.03	**^a^ 0.033**

AVBT: Anterior vertebral body tethering; NSF: Non-selective fusion; STF: Selective thoracic fusion; SD: Standard deviation; CHD: Coracoid Height Distance; SS: Sacral Slope; PT: Pelvic Tilt; PI: Pelvic Incidence; LL: Lumbar Lordosis; TK: Thoracic Kyphosis (T4-12); SVA: Sagittal Vertical Axis; CL: Cervical Lordosis; TPA: T1-Pelvic Angle; T1SPI: T1 Spinopelvic Inclination. ^a^ One-way ANOVA; ^b^ Kruskal–Wallis Test.

**Table 6:** The intragroup comparison of the preoperative, first postoperative, and final follow-up radiographic measurements of the AVBT group.

Variables	Preoperative ^1^	First Postoperative ^2^	Final Follow-Up ^3^	*p*
	Mean ± SD	Mean ± SD	Mean ± SD	
Major Cobb (°)	44.37 ± 6.61	9.42 ± 6.55	13.31 ± 7.21	**0.001 ^1–2, 1–3^**
CHD (mm)	16.33 ± 10.79	18.32 ± 10.93	10.29 ± 8.37	**0.028 ^2–3^**
SS (°)	39.67 ± 4.22	38.44 ± 8.96	36.27 ± 6.81	0.591
PT (°)	8.71 ± 9.24	10.17 ± 8.66	7.76 ± 10.26	0.089
PI (°)	48.5 ± 11.21	47.09 ± 16	42.27 ± 12.05	0.689
LL (°)	51.94 ± 7.32	51.41 ± 10.47	53.79 ± 13.58	0.635
TK (°)	31.25 ± 10.37	28.33 ± 6.18	34.08 ± 8.98	0.135
SVA (mm)	−3.08 ± 20	9.78 ± 25.12	2.79 ± 32.96	0.381
CL (°)	−1.37 ± 11.54	10.9 ± 6.48	11.82 ± 9.4	**0.001 ^1–2, 1–3^**
C7 Tilt (°)	−2.03 ± 2.43	3.48 ± 16.54	−3.58 ± 6.99	0.356
TPA (°)	3.74 ± 9.71	7.01 ± 8.84	4.6 ± 8.8	**0.042 ^1–2^**
T1SPI (°)	−3.25 ± 1.31	−2.65 ± 2.71	−3.98 ± 3.51	0.355

AVBT: Anterior vertebral body tethering; SD: Standard deviation; CHD: Coracoid Height Distance; SS: Sacral Slope; PT: Pelvic Tilt; PI: Pelvic Incidence; LL: Lumbar Lordosis; TK: Thoracic Kyphosis (T4-12); SVA: Sagittal Vertical Axis; CL: Cervical Lordosis; TPA: T1-Pelvic Angle; T1SPI: T1 Spinopelvic Inclination. Friedman’s Test.

**Table 7:** The comparison of the functional scores of the groups.

Variables	Group 1 (AVBT, n = 17)	Group 2 (NSF, n = 19)	Group 3 (STF, n = 15)	*p*
	Mean ± SD	Mean ± SD	Mean ± SD	
**SRS-22 Score**				
Pain	4.11 ± 0.71	4.07 ± 0.73	4.27 ± 0.39	0.918
Image	3.86 ± 0.65	3.49 ± 0.76	3.76 ± 0.85	0.298
Function	4.39 ± 0.69	4.33 ± 0.5	4.46 ± 0.56	0.586
Mental Health	3.56 ± 0.76	3.06 ± 0.81	3.49 ± 1.19	0.225
Satisfaction	4.11 ± 1.21	4.11 ± 0.81	4.32 ± 0.67	0.780
Total	3.99 ± 0.55	3.77 ± 0.52	4.02 ± 0.63	0.334
**ODI Score**	15.86 ± 15.85	17.29 ± 13.35	12.86 ± 13.96	0.635

AVBT: Anterior vertebral body tethering; NSF: Non-selective fusion; STF: Selective thoracic fusion; SD: Standard deviation; SRS-22: Scoliosis Research Society Score-22; ODI: Oswestry Disability Index. Kruskal–Wallis Test

## Data Availability

Due to privacy and ethical restrictions, the data are not publicly available.

## References

[1] Danielsson A.J., Romberg K., Nachemson A.L. (2006). Spinal range of motion, muscle endurance, and back pain and function at least 20 years after fusion or brace treatment for adolescent idiopathic scoliosis: A case-control study. Spine.

[2] Engsberg J.R., Lenke L.G., Reitenbach A.K., Hollander K.W., Bridwell K.H., Blanke K. (2002). Prospective evaluation of trunk range of motion in adolescents with idiopathic scoliosis undergoing spinal fusion surgery. Spine.

[3] Crawford C.H., Lenke L.G. (2010). Growth modulation by means of anterior tethering resulting in progressive correction of juvenile idiopathic scoliosis: A case report. J. Bone Jt. Surg. Am..

[4] Pehlivanoglu T., Oltulu I., Erdag Y., Korkmaz E., Sarioglu E., Ofluoglu E., Aydogan M. (2021). Double-sided vertebral body tethering of double adolescent idiopathic scoliosis curves: Radiographic outcomes of the first 13 patients with 2 years of follow-up. Eur. Spine J..

[5] Newton P.O., Parent S., Miyanji F., Alanay A., Lonner B.S., Neal K.M., Hoernschemeyer D.G., Yaszay B., Blakemore L.C., Shah S.A. (2022). Anterior Vertebral Body Tethering Compared with Posterior Spinal Fusion for Major Thoracic Curves: A Retrospective Comparison by the Harms Study Group. J. Bone Jt. Surg. Am..

[6] Buyuk A.F., Milbrandt T.A., Mathew S.E., Larson A.N. (2021). Measurable Thoracic Motion Remains at 1 Year Following Anterior Vertebral Body Tethering, with Sagittal Motion Greater Than Coronal Motion. J. Bone Jt. Surg. Am..

[7] Mathew S.E., Hargiss J.B., Milbrandt T.A., Stans A.A., Shaughnessy W.J., Larson A.N. (2022). Vertebral body tethering compared to posterior spinal fusion for skeletally immature adolescent idiopathic scoliosis patients: Preliminary results from a matched case–control study. Spine Deform..

[8] Trobisch P.D., Castelein R., Da Paz S. (2023). Radiographic outcome after vertebral body tethering of the lumbar spine. Eur. Spine J..

[9] Protopsaltis T.S., Lafage R., Smith J.S., Line B., Kim H.J., Hostin R., Ames C., Scheer J.K., Mundis G., Gupta M. (2023). The Importance of Sagittal Spinopelvic Alignment in Adult Spinal Deformity. Spine.

[10] Makhni M.C., Shillingford J.N., Laratta J.L., Hyun S.J., Kim Y.J. (2018). Restoration of Sagittal Balance in Spinal Deformity Surgery. J. Korean Neurosurg. Soc..

[11] Newton P.O., Kluck D.G., Saito W., Yaszay B., Bartley C.E., Bastrom T.P. (2018). Anterior Spinal Growth Tethering for Skeletally Immature Patients with Scoliosis: A Retrospective Look Two to Four Years Postoperatively. J. Bone Jt. Surg. Am..

[12] Yang X.A., David H.G., Gomez J.A. (2021). The importance of sagittal alignment in patients with adolescent idiopathic scoliosis and early onset scoliosis: A review on preoperative versus postoperative changes. Semin. Spine Surg..

[13] Baroncini A., Courvoisier A., Berjano P., Migliorini F., Eschweiler J., Kobbe P., Hildebrand F., Trobisch P.D. (2022). The effects of vertebral body tethering on sagittal parameters: Evaluations from a 2-years follow-up. Eur. Spine J..

[14] Cahill P.J., Miyanji F., Lullo B.R., Samdani A.F., Lonner B.S., Pahys J.M., Hwang S.W., Haber L.L., Alanay A., Shah S.A. (2024). Incidence of Tether Breakage in Anterior Vertebral Body Tethering. J. Pediatr. Orthop..

[15] Ök N., Büker N., Şavkın R., Bayrak G., Yörükoğlu A.Ç., Kıter A.E., Arık İ. (2019). Investigation of upper extremity functionality in adolescent patients with idiopathic scoliosis undergoing scoliosis surgery. South. Clin. Istanb. Eurasia.

[16] Fairbank J.C., Couper J., Davies J.B., O’Brien J.P. (1980). The Oswestry low back pain disability questionnaire. Physiotherapy.

[17] Fritz J.M., Irrgang J.J. (2001). A comparison of a modified Oswestry Low Back Pain Disability Questionnaire and the Quebec Back Pain Disability Scale. Phys. Ther..

[18] Baroncini A., Trobisch P.D., Birkenmaier C., Da Paz S., Migliorini F. (2022). Radiographic results after vertebral body tethering. Z. Orthop. Unfall..

[19] Mariscal G., Morales J., Pérez S., Rubio-Belmar P.A., Bovea-Marco M., Bas J.L., Bas P., Bas T. (2023). Meta-analysis on the efficacy and safety of anterior vertebral body tethering in adolescent idiopathic scoliosis. Eur. Spine J..

[20] Shin M., Arguelles G.R., Cahill P.J., Flynn J.M., Baldwin K.D., Anari J.B. (2021). Complications, reoperations, and mid-term outcomes following anterior vertebral body tethering versus posterior spinal fusion: A meta-analysis. JBJS Open Access.

[21] Hong J.Y., Suh S.W., Yang J.H., Park S.Y., Han J.H. (2013). Reliability analysis of shoulder balance measures: Comparison of the four available methods. Spine.

[22] Newton P.O., Bartley C.E., Bastrom T.P., Kluck D.G., Saito W., Yaszay B. (2020). Anterior Spinal Growth Modulation in Skeletally Immature Patients with Idiopathic Scoliosis: A Comparison with Posterior Spinal Fusion at 2 to 5 Years Postoperatively. J. Bone Jt. Surg. Am..

[23] Zhang H., Fan Y., Ni S., Pi G. (2022). The preliminary outcomes of vertebral body tethering in treating adolescent idiopathic scoliosis: A systematic review. Spine Deform..

